# Integrative Genetic Approach Facilitates Precision Strategies for Acute Myocardial Infarction

**DOI:** 10.3390/genes14071340

**Published:** 2023-06-26

**Authors:** Muzamil Khawaja, Rehma Siddiqui, Salim S. Virani, Christopher I. Amos, Dhrubajyoti Bandyopadhyay, Hafeez Ul Hassan Virk, Mahboob Alam, Hani Jneid, Chayakrit Krittanawong

**Affiliations:** 1 Department of Cardiology, Emory University School of Medicine, Atlanta, GA 30322, USA; 2 Department of Internal Medicine, The University of Mississippi Medical Center, Jackson, MS 39216, USA; 3 Department of Cardiology, The Aga Khan University, Karachi 74800, Pakistan; 4 Department of Cardiology, Baylor College of Medicine, Houston, TX 77030, USA; 5 Department of Medicine, Dan L Duncan Comprehensive Cancer Center, Baylor College of Medicine, Houston, TX 77054, USA; 6 Department of Cardiology, Westchester Medical Centre, New York Medical College, Valhalla, NY 10595, USA; 7 Department of Cardiology, University Hospitals Cleveland Medical Center, Case Western Reserve University, Cleveland, OH 44106, USA; 8 Department of Cardiology, The Texas Heart Institute, Baylor College of Medicine, Houston, TX 77030, USA; 9 Department of Cardiology, University of Texas Medical Branch, Houston, TX 77030, USA; 10 Department of Cardiology, NYU Langone Health and NYU School of Medicine, New York, NY 10016, USA

**Keywords:** acute myocardial infarction, genetics, precision medicine

## Abstract

Acute myocardial infarction remains a significant cause of mortality worldwide and its burden continues to grow. Its pathophysiology is known to be complex and multifactorial, with several acquired and inherited risk factors. As advances in technology and medical therapy continue, there is now increasing recognition of the role that genetics play in the development and management of myocardial infarction. The genetic determinants of acute coronary syndrome are still vastly understudied, but the advent of whole-genome scanning and genome-wide association studies has significantly expanded the current understanding of genetics and simultaneously fostered hope that genetic profiling and gene-guided treatments could substantially impact clinical outcomes. The identification of genes associated with acute myocardial infarction can help in the development of personalized medicine, risk stratification, and improved therapeutic strategies. In this context, several genes have been studied, and their potential involvement in increasing the risk for acute myocardial infarction is being investigated. As such, this article provides a review of some of the genes potentially related to an increased risk for acute myocardial infarction as well as the latest updates in gene-guided risk stratification and treatment strategies.

## 1. Introduction

**Figure 1:**
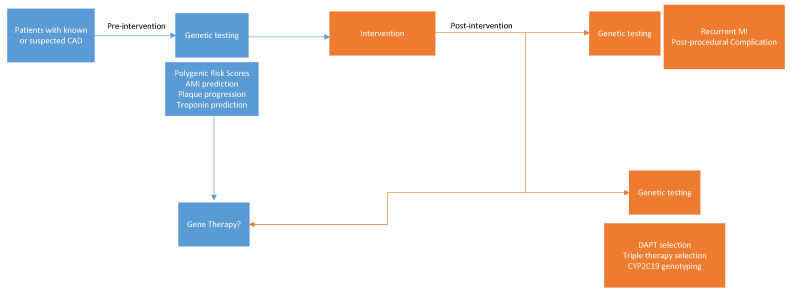
Potential applications for genetic evaluation in the prevention and treatment of myocardial infarction.

The definition of acute myocardial infarction (AMI) is broad, but this review will focus on the most utilized definition in the literature, which is Type 1 myocardial infarction. Type 1 myocardial infarction is secondary to atherosclerotic plaque disruption with varying degrees of atherosclerosis and thrombosis in the culprit lesion [1]. AMI is further characterized into non-ST segment elevation myocardial infarction and ST segment elevation myocardial infarction. These conditions share similar pathophysiological mechanisms, which involve ongoing disruption of blood flow to the myocardium. Because AMI is a major cause of morbidity and mortality worldwide, significant attention has been devoted to understanding its complex pathogenesis. Recent studies have raised the question of what role, if any, genetics play in its incidence. Unlike monogenetic diseases which follow a classic Mendelian pattern of inheritance, AMI remains a complicated disease process that is secondary to an interplay between environmental and genetic factors. However, recent data are emerging to support the use of personalized medicine via the use of genetic assessments. In fact, genetics and personalized medicine have been found to play an important role in the pharmacological management of coronary disease, and there are now signs of the advent of genetic risk stratification and profiling [2,3]. Personalized management of cardiovascular disease that is powered by genetics can facilitate risk prediction for incident or recurrent AMI, guide pharmacologic treatments, and even help with disease prevention and treatment with gene therapies (Figure 1). 

Such genetic evaluation depends on the ability to identify important genetic loci linked to the risk of AMI. However, to better comprehend why certain loci may increase the risk for AMI, it is crucial to first recognize its pathophysiology. The pathophysiology depends on a broad range of biological processes including lipid metabolism and atherosclerosis, platelet activation, angiogenesis, coagulation cascade, inflammation and endothelial dysfunction. The following sections will explore these pathophysiologies and discuss the literature behind the genes that have been implicated in such processes and subsequent risk for MI. For this review, the literature was surveyed using the PubMed search engine to identify contemporary articles on genes associated with AMI published between the years 2005 to 2023. Many loci have been previously identified as conferring risk for AMI, but the data have been inconsistent. While this review focuses on loci with stronger evidence in support of an association with AMI, there is always the possibility for future research to change current perspectives. The list of genetic loci discussed subsequently are listed in Table 1 with their mechanisms.

## 2. Atherosclerosis and Lipid Metabolism

Coronary atherosclerosis refers to the buildup of cholesterol, fat, calcium, and other substances to form plaque on the inner walls of coronary arteries. Several genetic loci have been identified to play a role in the formation and progression of coronary atherosclerosis to AMI.

One of the most important loci involved with atherosclerosis and possibly associated with AMI is that of *LDLR. LDLR* encodes for the low-density lipoprotein-receptor protein (LDL-R) [1]. Because low-density lipoprotein (LDL) transports cholesterol systemically, its functioning and serum levels can directly promote coronary atherosclerosis. The LDL-R protein plays a critical role in regulating LDL levels body through binding to LDL and removing it from circulation. Alterations in LDL-R functioning can theoretically contribute to coronary atherosclerosis and the risk for AMI. Studies by Do et al. and Tirdea et al. noted that carriers of certain alleles for *LDLR* had a higher risk for AMI [4,5]. In fact, Do et al. noted single allele carriers for *LDLR* had a 2.4-fold increased risk of AMI compared to controls while carriers of the *LDLR* null allele had an even higher risk (a 13-fold difference). This association was further substantiated in a study by Tajima et al. wherein targeted sequencing yielded 22 novel *LDLR* variants that showed a higher risk of AMI, higher LDL levels and younger age of onset for AMI in a Japanese population [6]. Pan-Lizcano et al. also identified several unique variants of *LDLR* associated with AMI in patients that had presented with ST-elevation myocardial infarction [7].

In addition to the *LDL-R* gene, *APO-A5* is involved with lipid metabolism and associated with a risk for AMI. *APO-A5* encodes for an apolipoprotein (ApoA-V) that is synthesized in the liver and found on the surface of triglyceride-rich lipoproteins such as very low-density lipoprotein, chylomicrons, and even high-density lipoprotein [8]. The protein interacts with lipoprotein lipase, an enzyme that breaks down triglycerides in the blood into free fatty acids that can be used for energy. ApoA-V’s primary function is to upregulate lipoprotein lipase [8]. This interaction alters plasma triglyceride levels and thereby affects the risk for atherosclerosis and AMI. For *APO-A5*, there are a handful of genetic variants identified, to this date, associated with an increased risk for AMI [9]. One study noted that a certain genotype was associated with greater risks for dyslipidemia and AMI [10]. In addition, Do et al. also observed that carriers of rare non-synonymous mutations at *APO-A5* had a 2.2-fold increased risk for AMI [4]. Their work also demonstrated that *APO-A5* mutation carriers had significantly higher plasma triglyceride levels than the control counterparts, suggesting a role for triglyceride metabolism in AMI. However, there may be mechanisms for APOA-V beyond triglyceride level regulation that factor into AMI incidence. This was purported by a study evaluating early-onset MI in an Italian population that observed a distinct *APO-A5* allele was strongly associated with AMI, independent of triglyceride level changes [11]. A separate study in the Chinese Han population identified two additional single nucleotide polymorphisms at *APO-A5* which conferred a higher risk of AMI [12].

Collectively, the number of genetic polymorphisms for *LDLR* and *APO-A5* potentially linked to AMI has continued to grow, and future studies may recognize the importance of these loci for other patient populations that have not been studied. While much is yet to be uncovered regarding the mechanisms and clinical implications for such polymorphisms, the potential for therapeutic targeting and possible risk stratification remains high.

## 3. Coagulation Cascade

The coagulation cascade plays a huge role in preventing as well as promoting thrombosis in the setting of AMI. Many loci for proteins involved in the coagulation cascade have been investigated for an association with AMI, but only one has been found to have consistently supportive data. One genetic locus that may play a major role in this process is the gene for plasminogen activator inhibitor-1 (*PAI-1*). PAI-1 is a protein that plays a critical role in inhibiting tissue-type plasminogen activator, which is responsible for dissolving thromboses [13]. A few genetic variants in *PAI-1* have now been recognized as linked to an increased risk of AMI.

The most extensively studied *PAI-1* variant is the 4G/5G polymorphism. The 5G allele involves the insertion of five guanine nucleotides in the promoter sequence of *PAI-1*, while a single guanine deletion results in the 4G allele [14]. The 4G allele is associated with higher PAI-1 levels than the 5G allele, leading to impaired fibrinolysis and an increased risk of thrombosis. The 4G allele is reported to increase the risk for atherosclerosis and coronary artery disease, while the 5G allele may increase the risk of abdominal aortic aneurysm [15,16].

Numerous studies have investigated the association between the 4G and 5G polymorphisms and AMI risk, with inconsistent results. More recently, however, a large Mendelian randomization meta-analysis noted that the *PAI-1 4G* allele slightly increases the risk for AMI [17]. This was replicated by Morange et al., who demonstrated certain *PAI-1* haplotypes were mildly associated with plasma levels of PAI-1 and with the risk of AMI in nonsmokers [18]. In addition, a retrospective study of patients that had suffered acute coronary occlusion found that only the 4G/5G genotype was associated with coronary artery occlusion [19]. In parallel, Kumar et al. retrospectively evaluated 100 patients with AMI and chronic stable angina in India and found the frequencies of 4G/5G genotypes were significantly higher in AMI cases than in controls [20]. In addition, there was a significant relationship between the 4G/5G polymorphism and AMI risk under the dominant and codominant genotype. This was also noted in a study of Egyptian patients wherein the authors concluded that the 4G/4G genotype and 4G allele of the *PAI-1* gene were associated with the risk of AMI and its morbidity [21]. The PAI-1 4G/4G genotype was also interestingly associated with mortality due to AMI. 

The increased risk of AMI associated with the 4G allele is thought to be mediated by impaired fibrinolysis, leading to the formation of thrombi that can occlude coronary arteries. Additionally, the 4G allele has been associated with other risk factors for AMI, such as hypertension, hyperlipidemia, and diabetes [22,23,24].

The effect of the 4G/5G polymorphism on AMI risk may vary depending on other genetic and environmental factors. For example, a study of Finnish men found that the increased risk of AMI associated with the 4G allele was limited to individuals with a family history of premature AMI [25].

Overall, the available evidence suggests that the *PAI-1* 4G/5G polymorphism may be a genetic risk factor for AMI. However, more studies are needed to confirm this association and to investigate the underlying mechanisms linking *PAI-1* gene variants to AMI risk. Although there are currently no other identified coagulation pathway polymorphisms conferring a significant risk for AMI, these findings for *PAI-1* may have implications for the future development of personalized prevention and treatment strategies based on genetic risk factors.

## 4. Endothelial Function

Abnormal regulation of vascular smooth muscle proliferation is an important factor that can often lead to atherosclerosis. Cell proliferation depends significantly on the sequential activation of cyclin and cyclin-dependent kinase complexes [26]. These complexes are then subject to regulation through reversible association with cyclin-dependent kinase inhibitory proteins (CKIs). Variation in expression and activity of these CKIs can thereby influence the response of vascular smooth muscle cells to mechanical stimuli (such as blood flow) and to molecules (such as nitric oxide, interleukins, angiotensin II, and growth factors) to ultimately alter the risk of coronary atherosclerosis and AMI.

One such CKI is encoded for by the *CDKN1A* gene. Rodriguez et al. studied the frequency of various *CKDN1A* polymorphisms in patients with coronary disease and myocardial infarction. They found a guanine/thymine repeat sequence in the promoter region of the *CDKN1A* gene that occurred with significantly higher frequency in patients that had suffered AMI [26].

However, it is worth noting that data on CKI genes and the correlation with incident AMI is extremely limited at this time. Additional genes related to endothelial dysfunction have been investigated but no others have been consistently associated with AMI risk. Further research is necessary to better elucidate the association of *CDKN1A*, if any, with the risk for AMI and to possibly identify additional endothelial function genetic polymorphisms conferring a higher risk for AMI.

## 5. Myocyte Stability

Glycine is an important amino acid that may play a significant protective role against cardiovascular disease. It has been reported to protect against ischemia-reperfusion injury in cells through inhibiting apoptosis. Glycine receptors in the myocardial membrane, therefore, have become a potential target for genetic investigation aimed at reducing the risk for AMI. The glycine receptor α2 has more recently been identified as a locus significantly associated with AMI in a microarray analysis study by Yang et al. [27]. The mechanisms by which this locus may promote AMI are still unknown but additional functional studies on this receptor may be worth pursuing.

Additional loci of interest for AMI risk are those encoding for A-kinase-anchoring proteins (AKAPs). AKAPs are scaffolding proteins that regulate the cellular cyclic adenosine monophosphate response [27]. Several AKAPs are reported to be expressed in the heart and participate in cardiovascular functions through various mechanisms. For example, AKAPs help anchor protein kinase A in the sarcomere for the phosphorylation of myofibril proteins in contractile response [27]. In addition, AKAPs can regulate cardiac contractility, rhythm, and even remodeling [28,29,30]. Recently, AKAP12 was found to be significantly associated with AMI [27]. Previously, AKAP 12 had been primarily associated with various cellular functions, including cytoskeletal architecture and cell cycle regulation, but its potential role in AMI may be plausible given the variety of cardiac functions of other AKAPs.

## 6. Genetic Risk Stratification for Acute Myocardial Infarction

### Polygenic Risk Scores and Utility of Genetic Scores for AMI

Cardiovascular disease remains the leading cause of death worldwide, and now more than ever, there is a growing focus on prevention. Prevention of cardiovascular disease and AMI has evolved significantly over the past few decades, but risk prediction has remained quite imprecise given the complexity of risk factors for AMI. Of course, identification of traditional patient risk factors such as smoking status, obesity, or history of diabetes or hyperlipidemia can help identify patients earlier as high risk. While the recent 2021 American Heart Association/American College of Cardiology (AHA/ACC) Guidelines for management of chest pain recommend the use of institution-based accelerated diagnostic protocols to guide efficient and effective management of AMI patients, there are still areas of improvement [31]. In fact, while current protocols have significantly improved door-to-balloon times for patients with AMI, there are still important delays from symptom onset to presentation that may be addressed through earlier risk stratification with genetic assessments [31]. Given the strong aggregation of coronary disease in families, genetics have long been expected to influence a patient’s risk for AMI. To build on this further, genetic assessment may offer an earlier and more personalized prevention strategy. The latest genetic scoring system under investigation is the polygenic risk score (PRS). A PRS is a statistical tool that uses genetic information to predict one’s risk of developing a certain disease or condition. The score is a sum of all the effects of various genetic variants associated with disease risk. These include genes such as the previously discussed loci and many others that may contribute to a patient’s individual risk for disease development. The PRS can be used in conjunction with other risk factors, such as age, sex, and lifestyle, to help guide decisions about prevention and treatment options. It is used to estimate the cumulative sum of the disease risk conferred by different disease-associated single nucleotide polymorphisms (SNPs) within a genome [32]. This score requires a list of SNPs and their expected effects, often obtained from a genome-wide association study. At this time, the PRS is not fully incorporated into clinical practice, but it is notably garnering recognition as a useful predictive tool. Although the ACC/AHA recommends calculating the 10-year cardiovascular risk for all patients aged 40 to 75 years with the atherosclerotic cardiovascular disease risk calculator [33], studies suggest that the addition of the PRS to the AHA/ACC atherosclerotic cardiovascular disease risk calculator improves prediction of cardiovascular events [34,35,36,37,38]. While PRSs may prove useful for risk assessment of AMI, few have been studied and validated in the literature. Manikpurage et al. studied a PRS weighing the effects of over 1 million SNPs associated with coronary artery disease and found it to be a significant predictor of AMI incidence and all-cause mortality [39]. Brautbar et al. similarly developed a PRS consisting of carefully selected SNPs associated with coronary disease [40]. This PRS, when used in combination with the Atherosclerosis Risk in Communities score, significantly improved reclassification of patients to predict major cardiovascular outcomes. A separate study on the PRS for coronary disease was applied to a population of Korean patients who had suffered early-onset AMI. The patients with early AMI had significantly higher PRSs than the control cohort and a higher PRS correlated with a higher incidence of revascularization [41]. However, it is important to recognize the various limitations of PRSs. The first is that early genetic studies on AMI, many of which drive the PRSs studied today, had primarily focused on individuals of European ancestry, ultimately limiting their generalizability [42]. Secondly, PRSs have largely been developed and validated for coronary artery disease but have not been as extensively studied for AMI. Another limitation is that although PRSs can estimate a patient’s residual risk beyond traditional risk factors, the mechanisms to explain such genetic effect modification are largely unclear. Additional studies are needed to further identify any novel pathways or mechanisms for such effects to help guide treatment. There is also the issue of cost-effectiveness for PRSs, which continues to be understudied. Lastly, the most effective PRS screening technology and target populations remain to be defined. PRSs have the potential to identify patients at risk for AMI much sooner than other standard stratification tools, but there is still a significant need for further investigation into PRSs before they can be clinically recommended and applied.

## 7. Gene Therapy for Acute Myocardial Infarction

While risk stratification and conventional therapies, such as revascularization and pharmacological interventions, have significantly improved patient outcomes, there remains a need for novel therapeutic strategies. Gene therapy, the transfer or modulation of specific genes to correct or modulate disease-related pathways, holds great promise in addressing the underlying causes of AMI. While a significant amount of research is ongoing in the realm of gene therapy for AMI, there is some preliminary evidence in animal models to support the use of gene transfer, gene editing, and gene silencing approaches (Table 2).

Gene transfer refers to the technique of integrating new genetic material into a genome to treat or prevent disease. Various viral and non-viral vectors have been employed for gene transfer in AMI. Adeno-associated viruses, lentiviruses, and adenoviruses are commonly used viral vectors due to their high transduction efficiency [43]. Non-viral vectors such as liposomes and nanoparticles offer advantages such as reduced immunogenicity and improved safety profiles [44]. There are several target transfer genes for AMI that have been investigated to date, but only a few are discussed subsequently. Vascular endothelial growth factor (*VEGF*) gene transfer aims to promote angiogenesis and improve blood supply to ischemic myocardium. Preclinical studies have demonstrated the efficacy of *VEGF* gene therapy in promoting neovascularization and reducing infarct size in animal models [45,46,47]. Studies in human models are still ongoing. A separate target for gene transfer in AMI treatment is the stromal cell-derived factor-1 (*SDF-1*) gene. *SDF-1* gene therapy enhances stem cell recruitment and homing to the injured heart to induce cardiac repair and regeneration. Animal studies have shown improved cardiac function and reduced scar formation in the setting of AMI following SDF-1 gene therapy [48], but human studies are warranted. Another target for AMI treatment via gene transfer is the Hepatocyte growth factor (*HGF*) gene. *HGF* gene therapy promotes cardiomyocyte survival, angiogenesis, and tissue repair. Preclinical studies have demonstrated improved cardiac function and reduced infarct size after *HGF* gene transfer. In fact, Kondo et al. trialed a novel strategy for gene transfer using ultrasonic microbubble destruction to deliver *HGF* to treat AMI in rats. They found that *HGF* gene transfer via this mechanism effectively enhanced angiogenesis, limited infarction size and prevented left ventricular remodeling after AMI [49]. While these are just a few of the genes targeted with gene transfer for AMI treatment, the potential remains high for this genetic therapy to improve AMI treatment strategies.

Gene editing is an alternative gene-based therapy to gene transfer that holds promise for the treatment and even prevention of AMI. One specific form of gene editing involves the utilization of clustered regularly interspaced short palindromic repeats (CRISPR)-Cas (CRISPR-associated) 9 technology. CRISPR-Cas9 technology allows precise modification of the genome, enabling the correction of disease-causing genetic mutations associated with AMI [50]. Gene editing using CRISPR-Cas9 even holds potential for the treatment of inherited cardiac disorders, such as familial hypercholesterolemia, which predisposes individuals to AMI. However, it is worth noting the challenges with CRISPR-Cas9 gene editing that include difficulties with delivery of the system and accurate genetic targeting [50]. Other novel genetic technologies for gene editing include zinc finger nucleases (ZFNs) and transcription activator-like effector nucleases (TALENs). These gene editing tools also facilitate targeted genetic modifications [51]. Studies using ZFNs and TALENs have shown successful gene editing in animal models of AMI, targeting genes associated with cardiac hypertrophy and fibrosis [52,53]. However, human studies are still ongoing.

Another significant area of genetic therapy focuses on gene silencing. One such method of silencing utilizes small interfering RNAs (siRNAs). SiRNA-based therapies can silence specific genes involved in AMI pathogenesis. Targeting pro-inflammatory cytokines, such as tumor necrosis factor-α, has shown promise in preclinical studies via reducing inflammation and improving cardiac function [54,55,56]. Antisense oligonucleotides are also a growing area of interest as they too can modulate gene expression through silencing mRNA sequences. Antisense oligonucleotides targeting genes involved in lipid metabolism, such as apolipoprotein-B, have demonstrated potential in reducing atherosclerosis and the risk of AMI [57,58,59].

## 8. Challenges and Future Directions for Genomics in Acute Myocardial Infarction

### 8.1. Risk Stratification

As previously mentioned, genetic profiling and risk stratification for AMI have the potential to revolutionize the prevention, diagnosis, and treatment of this cardiovascular event. However, several challenges exist in implementing these strategies effectively. Firstly, AMI is clearly a multifactorial disease influenced by both genetic and environmental factors. To this date, very few genetic loci have been identified as potentially contributing to AMI risk, but such associations are difficult to isolate because of the complex interactions between multiple genes and environmental factors. Furthermore, the influence of genetic variations on disease development may vary among different populations, making it difficult to generalize findings across diverse populations.

The introduction of PRSs has shown promise in predicting the risk of AMI, but their clinical utility and accuracy for individual risk prediction are still being evaluated. Developing standardized and validated PRSs that can reliably predict an individual’s risk of AMI remains a challenge.

In addition, the functional implications of many genetic variants linked to AMI are still unclear. Understanding how these variations affect biological pathways and contribute to AMI development is crucial for effective risk stratification and targeted interventions. However, functional characterization of these variants requires extensive experimental studies and large-scale functional genomics efforts.

Additional barriers to applying genetic risk stratification are ethical and privacy concerns. Access to an individual’s genetic information can provide valuable insights into their individual risks, but this information is highly sensitive and raises concerns related to data security, confidentiality, and potential discrimination. Addressing these ethical and privacy concerns is essential to ensure the responsible use of genetic profiling in risk stratification.

Lastly, there are issues with cost and accessibility. Genetic testing and analysis can be expensive, limiting its availability to certain populations. Additionally, the interpretation and translation of genetic data into clinical practice require specialized expertise, which may not be readily available in all healthcare settings. Efforts to reduce costs, increase accessibility, and develop user-friendly tools for genetic risk stratification are necessary to overcome these challenges.

Despite these challenges, ongoing advancements in genomic research, technology, and data analysis methods hold promise for overcoming these limitations. Collaborative efforts among researchers, clinicians, policymakers, and regulatory bodies are crucial to address these challenges, validate the clinical utility of genetic profiling, and develop personalized strategies for risk stratification and prevention of AMI.

### 8.2. Gene Therapy

There are also several barriers to the clinical application of gene therapies for AMI. One of the key challenges in gene therapy for AMI is the efficient and targeted delivery of therapeutic genes to the myocardium. Research efforts should focus on developing more efficient viral and non-viral vectors with enhanced transduction efficiency and tissue specificity. Further optimization of vector design, such as modifying viral capsids or developing tissue-specific promoters, can improve gene delivery to the heart, ensuring higher levels of transgene expression. In addition, improved targeting strategies are needed to maximize the therapeutic efficacy of gene therapy for AMI. Researchers should explore innovative approaches, such as cardiac-specific ligands, antibodies, or nanoparticles, to enhance the specificity and selectivity of gene delivery to the ischemic myocardium. Combining imaging modalities, such as magnetic resonance imaging or positron emission tomography, with gene therapy can enable real-time monitoring of gene expression and therapeutic outcomes. There is also the issue of gene therapies having unintended off-target effects, leading to adverse events or disruptions in normal gene function. Future research should focus on developing more precise gene editing technologies, such as CRISPR-Cas9, with improved specificity and reduced off-target effects. Robust preclinical studies and comprehensive safety assessments are necessary to ensure the long-term safety and efficacy of gene therapies for AMI.

Even with improved safety profiles for gene therapy, identifying novel therapeutic targets is crucial for advancing gene therapy in AMI. Researchers should continue to investigate the roles of various genes and molecular pathways involved in AMI pathogenesis to identify potential targets for gene therapies. For example, targeting genes associated with inflammation, oxidative stress, apoptosis, or fibrosis could offer new therapeutic avenues for preventing or mitigating AMI. Lastly, while preclinical studies have shown promising results, large-scale clinical trials are necessary to evaluate the safety, efficacy, and long-term effects of gene therapies for AMI. These trials should assess clinical outcomes, such as reduction in infarct size, improvement in cardiac function, and long-term survival rates. Furthermore, studies should also investigate the potential of combining gene therapy with other treatment modalities, such as revascularization procedures or pharmacological interventions, to achieve synergistic effects.

It is also worth mentioning that the realm of cardiovascular genomics for AMI may likely see the advent of robust artificial intelligence technologies to better guide prognostic and therapeutic strategies. Recent reviews on the current state of artificial intelligence note its various uses such as the application of deep learning models and novel techniques to eliminate data redundancy, improve outcome prediction, identify novel genes, understand the contribution of noncoding mutations, and recognize epigenetic and intergenic interactions [60,61]. However, while these potential applications are under development, there are notable limitations to clinical implementation for AMI risk assessment and management. These include, but are not limited to, the lack of robust knowledge of cardiovascular genomics, the difficulty of generalized application, and the uncertainty regarding how to best incorporate such technologies into clinical workflow. Among currently practicing physicians, there is also a lack of understanding and recognition of the potential utility for these tools. Nevertheless, the world of artificial intelligence genomics will continue to grow and may very well drastically alter the management approach for AMI patients.

## 9. Conclusions

AMI is a complex disease process, involving many factors that contribute to its overall development. As discussed, the role of genetics in AMI is better understood today, but there is still much to be learned regarding the intricate and multifaceted interactions between genes and proteins, environmental factors, and patient factors specific to each individual that all collectively contribute to phenotypic expression. As mentioned previously, the number of genetic loci potentially linked to AMI incidence across diverse world populations is continually expanding, but insight into their exact mechanisms and relevance for prevention is still warranted. There is a dire need to devote more effort into understanding these complex relationships to better risk-stratify such patients. Moreover, gene therapy holds significant promise for the prevention and treatment of AMI. However, to realize its full potential, future research should focus on improving gene delivery systems, enhancing targeting strategies, addressing off-target effects, exploring novel therapeutic targets, and conducting large-scale clinical trials. Advancements in understanding the genetics behind AMI can further guide the world of personalized cardiovascular medicine and ultimately contribute to the development of safer, more effective gene therapies, ultimately reducing the burden of AMI and improving patient outcomes.

## Figures and Tables

**Table 1:** Gene loci involved with AMI.

Cellular Process	Gene	Normal Function of Protein
Lipid Metabolism	*LDLR*	Clearance of LDL from circulation via endocytosis
Lipid Metabolism	*APO-A5*	Upregulation of lipoprotein lipase
Coagulation Cascade	*PAI-1*	Inhibition of tissue plasminogen activator
Endothelial Function	*CDKN1A*	Regulation of vascular smooth muscle cell proliferation
Myocyte Stability	*GLAR2*	Unknown
Myocyte Stability	*AKAP*	Regulation of cardiac contractility, remodeling and structural scaffolding

**Table 2:** Gene Therapies for AMI.

Therapy	Mechanism	Examples
Gene Transfer	Integrating new genetic material into a genome to treat or prevent disease via the use of viral and non-viral vectors	VEGF gene transfer to promote angiogenesis and improve blood supply to ischemic myocardium;
		SDF-1 gene transfer enhances stem cell recruitment and homing to the injured heart to induce cardiac repair and regeneration;
		HGF gene transfer promotes cardiomyocyte survival, angiogenesis, and tissue repair
Gene Editing	Altering the genome using CRISPR-Cas9, zinc finger nucleases, and transcription activator	CRISPR-Cas9 allows precise modification of the genome, enabling the correction of disease-causing genetic mutations associated with MI
Gene Silencing	Silencing genes via the use of siRNAs	SiRNAs to target pro-inflammatory cytokines to reduce inflammation and improve cardiac function;
		Antisense oligonucleotides targeting genes involved in lipid metabolism, such as apolipoprotein-B

## Data Availability

No new data were created or analyzed in this study. Data sharing is not applicable to this article.
